# The Clock Mechanism Influences Neurobiology and Adaptations to Heart Failure in *Clock*^*∆19/∆19*^ Mice With Implications for Circadian Medicine

**DOI:** 10.1038/s41598-019-41469-7

**Published:** 2019-03-21

**Authors:** Austin T. H. Duong, Cristine J. Reitz, Emma L. Louth, Samantha D. Creighton, Mina Rasouli, Ashley Zwaiman, Jeffrey T. Kroetsch, Steffen-Sebastian Bolz, Boyer D. Winters, Craig D. C. Bailey, Tami A. Martino

**Affiliations:** 1 Centre for Cardiovascular Investigations, Biomedical Sciences, University of Guelph, Guelph, Ontario Canada; 2 Department of Psychology, University of Guelph, Guelph, Ontario Canada; 3 Department of Physiology, University of Toronto, Toronto, Ontario Canada

## Abstract

In this study we investigated the role of the circadian mechanism on cognition-relevant brain regions and neurobiological impairments associated with heart failure (HF), using murine models. We found that the circadian mechanism is an important regulator of healthy cognitive system neurobiology. Normal *Clock*^*∆19/∆19*^ mice had neurons with smaller apical dendrite trees in the medial prefrontal cortex (mPFC), and hippocampus, showed impaired visual-spatial memory, and exhibited lower cerebrovascular myogenic tone, versus wild types (WT). We then used the left anterior descending coronary artery ligation model to investigate adaptations in response to HF. Intriguingly, adaptations to neuron morphology, memory, and cerebrovascular tone occurred in differing magnitude and direction between *Clock*^*∆19/∆19*^ and WT mice, ultimately converging in HF. To investigate this dichotomous response, we performed microarrays and found genes crucial for growth and stress pathways that were altered in *Clock*^*∆19/∆19*^ mPFC and hippocampus. Thus these data demonstrate for the first time that (i) the circadian mechanism plays a role in neuron morphology and function; (ii) there are changes in neuron morphology and function in HF; (iii) CLOCK influences neurobiological gene adaptations to HF at a cellular level. These findings have clinical relevance as patients with HF often present with concurrent neurocognitive impairments. There is no cure for HF, and new understanding is needed to reduce morbidity and improve the quality of life for HF patients.

## Introduction

Ischemic heart disease leading to myocardial infarction (MI, heart attack) and heart failure (HF) is a leading cause of morbidity and mortality worldwide^1^. Cognitive impairment, depression or brain changes are frequently observed in patients with HF^2–4^. Here we investigated a role for the circadian mechanism in neurocognitive impairments in HF, using murine models. In support of this approach, recent experimental and clinical studies show that the circadian mechanism is important for cardiovascular physiology. It underlies daily rhythms in heart rate^5^, blood pressure^6^, and organizing of the cardiac genome^7–14^, proteome^15–17^, contractility and metabolism (reviewed in^11,18–20^). Moreover, we and others have shown that the circadian mechanism contributes to remodeling in ischemic heart disease and HF^9,12,21–32^. However, how the circadian mechanism affects the neurobiological adaptations in HF is not known, in large part because studies have focused only on the heart and have not investigated what happens concurrently in the brain.

The circadian system coordinates our physiology with the diurnal environment – mammals are awake in the day or at night (reviewed in^33,34^). Briefly, the system is hierarchically orchestrated; time-setting light cues (Zeitgebers, ZT) are received by the master clock in the suprachiasmatic nucleus (SCN) of the hypothalamus. The SCN then communicates via neural and hormonal outputs to all other regions of the brain and body to set time. At a molecular level, the circadian mechanism is comprised of a 24-hour transcription and translation loop present in virtually all our cells. CLOCK is a key component of the circadian mechanism and relative abundance over the 24-hour daily cycles drives the positive and negative arms of the circadian mechanism^35,36^. In doing so, CLOCK regulates daily patterns of genes and proteins in the normal heart, and cardiac remodeling in heart disease (e.g.^10,16,21,22,37^). However, whether CLOCK also has an effect on the brain in response to HF is largely unknown.

Here we investigate the role of the circadian mechanism on normal neurobiology using *Clock*^*∆19/∆19*^ mice, and how the circadian mechanism contributes to neurobiological adaptations within cognition-relevant brain regions in HF. We use the clinically relevant murine left anterior descending coronary artery ligation (MI) model, to simulate the possible effects on the evolution of HF in humans^23,24^. We demonstrate that *Clock*^*∆19/∆19*^ mice have neurons with smaller apical dendrite trees within layer 2/3 of the medial prefrontal cortex (mPFC) and CA1 region of the hippocampus, and deficits in visual-spatial memory, compared with wild type (WT) mice. Interestingly, in response to HF, the *Clock*^*∆19/∆19*^ mice exhibit reduced cardiac remodelling and better outcomes. Moreover, the *Clock*^*∆19/∆19*^ mice show differences in the magnitude and direction of their neurobiological responses, as compared to WTs. However, despite different neurobiological adaptations, they result in similar end outcomes in terms of neuron morphology, memory, and cerebrovascular tone in HF. To investigate the mechanisms underlying this dichotomous response, we examined gene expression at baseline, MI and HF in the mPFC and hippocampus in *Clock*^*∆19/∆19*^ vs. WT mice. We found differential activation of genes important for neural growth, cytoskeleton, signalling and metabolism. Collectively these data reveal that CLOCK is an important regulator of healthy cognitive system neurobiology, and adaptations in neuron morphology and function in HF. Importantly, there is no cure for HF, and neurocognitive impairments frequently coincide in patients with HF. Elucidating a role for the circadian mechanism in the neurobiology of the cognitive system can lead to new strategies to reduce morbidity and improve the quality of life of patients with HF.

## Results

### Characterization of circadian *Clock*^*∆19/∆19*^ vs. WT models

**Figure 1:**
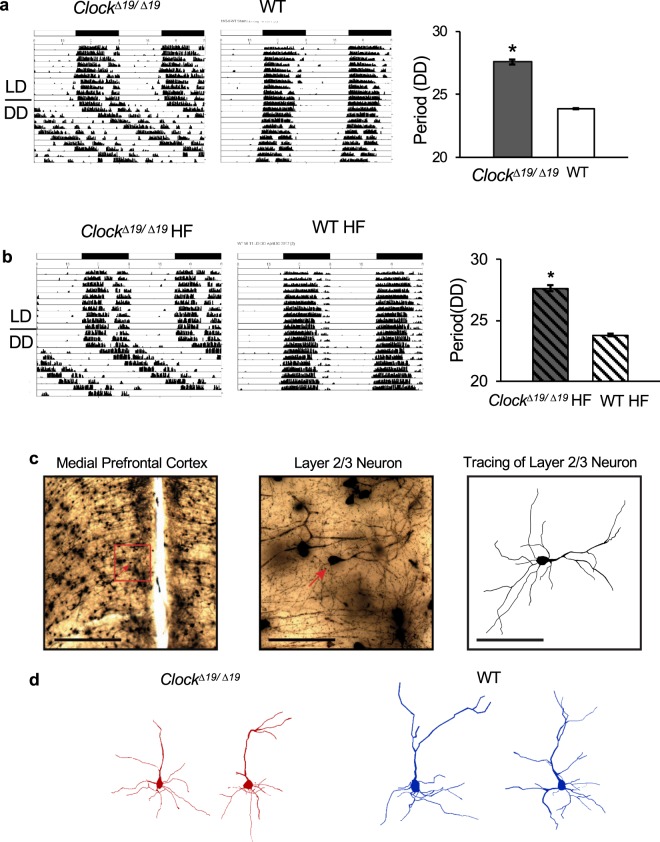
Murine *Clock*^*∆19/∆19*^ and WT models. (**a**) Wheel running actigraphy characterizing *Clock*^*∆19/∆19*^ (left) and WT (middle) mice under normal diurnal conditions (12 hour light:12 hour dark) for 10 days, and circadian (constant darkness, DD) conditions for 10 days. Quantification of period under DD (right) showing that the CLOCK mutation extends the circadian period from 23.9 hours to ~27.6 hours, as anticipated (n = 4 mice/group, *P* < 0.05). (**b**) Actigraphy of HF mice, showing that *Clock*^*∆19/∆19*^ (left) and WT (middle) mice, and period under DD (right) maintain their respective phenotypes, and are significantly different from each other (n = 4 mice/group, *P* < 0.05). (**c**) Exemplar photomicrographs of one traced neuron are shown. A low-magnification view is shown in the left panel, with the neuron of interest indicated by the red arrow and a scale bar of 500 µm. A high-magnification view of the area enclosed by the red box is shown in the middle panel, with a scale bar of 100 µm, and a tracing of a layer 2/3 neuron on the right, with a scale bar of 100 µm. (**d**) Representative neuron tracings illustrating the smaller mPFC apical dendrite tree size in *Clock*^*∆19/∆19*^ versus WT mice. *Indicates *P* < 0.05 by Bonferroni *post-hoc* analysis.

We postulated that the circadian mechanism influences neurobiology in the brain’s cognitive systems. To test this, we focused on the circadian factor CLOCK, as it is a canonical part of the circadian mechanism, and relatively well characterized in experimental heart disease models. *Clock*^*∆19/∆19*^ mice were genotyped and phenotyped using conventional approaches. The *Clock*^*∆19/∆19*^ mutation extends the circadian period from ~23.9 hours in WT mice to ~27.6 hours in *Clock*^*∆19/∆19*^ mice. Figure 1a shows representative wheel running actigraphy for *Clock*^*∆19/∆19*^ (left) and WT (middle) mice under a diurnal 12 hour light: 12 hour dark (LD) cycle and constant darkness (DD), and quantification of period under DD (right, p < 0.0001, n = 4 per group).

Next, in order to investigate the role of the circadian mechanism in the neurobiological adaptations in the brain’s cognitive systems in heart failure (HF), we subjected *Clock*^*∆19/∆19*^ and WT mice to left anterior descending (LAD) coronary artery ligation (HF model). Mice housed under normal diurnal or circadian conditions maintain their wheel running behaviour, as anticipated. As shown in Fig. 1b, the *Clock*^*∆19/∆19*^ mice maintain a period of ~27.6 hours, and WT mice maintain a period of ~23.8 hours, consistent with the periods observed in controls (Fig. 1a), suggesting that the HF model does not alter the circadian mechanism.

We then characterized the pathophysiology of our murine HF model, using classic echocardiography and *in-vivo* hemodynamics approaches. As shown in Table 1, both *Clock*^*∆19/∆19*^ and WT mice exhibit adverse cardiac remodeling, including an increase in heart size and reduced function, by 1 week post-infarction. Moreover, the evolution of structural and functional pathophysiology by 8 weeks is characteristic of HF. This includes significantly (P < 0.05) enlarged hearts, with increased left ventricular internal diastolic (LVIDd) and systolic (LVIDs) dimensions, reduce % ejection fraction (%EF) and decreased fractional shortening (%FS) by echocardiography. Moreover, both genotypes develop significant (P < 0.05) hypotension, and dP/dt max/min are decreased, by *in vivo* pressure-volume hemodynamics. We observed that cardiac remodeling is less in the *Clock*^*∆19/∆19*^ versus WT mice; however, both genotypes show significant remodeling and development of HF. Thus this model triggers structural and functional ventricular remodeling leading to HF, and serves as a template to investigate what happens in the brain in HF.

**Table 1:** Cardiac structure and function by echocardiography and *in vivo* hemodynamics.

Echocardiography (n)	*Clock*^Δ19/Δ19^ HF mice	WT HF mice	*Clock*^Δ19/Δ19^ Controls	WT Controls
	8	8	5	5
**Baseline**				
LVIDd (mm)	3.95 ± 0.03	3.95 ± 0.02	3.97 ± 0.03	3.95 ± 0.02
LVIDs (mm)	2.32 ± 0.02	2.35 ± 0.02	2.38 ± 0.03	2.33 ± 0.01
EF (%)	78.39 ± 0.40	77.42 ± 0.48	76.98 ± 0.57	78.10 ± 0.37
FS (%)	41.28 ± 0.39	40.37 ± 0.43	40.02 ± 0.51	41.01 ± 0.34
**1 week post-infarction**				
LVIDd (mm)	4.94 ± 0.08*	5.11 ± 0.16^§^	4.03 ± 0.02	3.98 ± 0.03
LVIDs (mm)	3.69 ± 0.09*	3.87 ± 0.15^§^	2.43 ± 0.03	2.38 ± 0.05
EF (%)	56.55 ± 1.58*	54.76 ± 1.97^§^	76.50 ± 0.51	77.26 ± 1.02
FS (%)	25.40 ± 0.94*	24.50 ± 1.15^§^	39.61 ± 0.45	40.25 ± 0.90
**8 week - HF**				
LVIDd (mm)	5.09 ± 0.08*^†^	5.65 ± 0.11^§^	4.05 ± 0.03	4.00 ± 0.03
LVIDs (mm)	3.81 ± 0.09*^†^	4.45 ± 0.11^§^	2.46 ± 0.03	2.39 ± 0.03
EF (%)	56.37 ± 1.17*^†^	49.42 ± 1.08^§^	76.08 ± 0.32	77.11 ± 0.48
FS (%)	25.38 ± 0.68*^†^	21.52 ± 0.57^§^	39.23 ± 0.24	40.11 ± 0.48
**Hemodynamics (HF)**				
LVESP (mmHg)	88.2 ± 1.5*	87.0 ± 0.8^§^	97.9 ± 1.4	98.4 ± 0.8
LVEDP (mmHg)	3.5 ± 1.5*	2.5 ± 1.1	−0.4 ± 0.4	0.4 ± 0.7
LVESV (μl)	23.5 ± 1.0*^†^	36.4 ± 1.4^§^	9.8 ± 0.9	10.6 ± 0.4
LVEDV (μl)	46.4 ± 0.6*^†^	54.7 ± 1.3^§^	34.7 ± 1.4	35.9 ± 1.1
SV (μl)	22.9 ± 0.6^†^	18.3 ± 0.5^§^	24.0 ± 0.6	24.1 ± 1.0
CO (mL/min)	11.5 ± 0.4^†^	9.6 ± 0.5^§^	12.7 ± 0.6	13.3 ± 0.8
dP/dt_max_ (mmHg/sec)	6939 ± 394*	5959 ± 258^§^	10420 ± 674	9744 ± 864
dP/dt_min_ (mmHg/sec)	5905 ± 458*	5278 ± 258^§^	9403 ± 1030	9830 ± 966
SBP (mmHg)	82.9 ± 1.0*^†^	89.3 ± 0.9^§^	97.8 ± 1.2	98.1 ± 1.0
DBP (mmHg)	58.7 ± 0.7*	60.6 ± 0.5	64.3 ± 1.4	63.6 ± 0.6
MAP (mmHg)	66.3 ± 0.7*^†^	69.7 ± 0.3^§^	74.7 ± 0.9	74.3 ± 0.3
**Morphometry (HF)**				
HW (mg)	158 ± 2*	162 ± 6^§^	140 ± 3	130 ± 3
HW:BW (mg/g)	5.03 ± 0.10*^†^	5.86 ± 0.25^§^	4.43 ± 0.15	4.14 ± 0.12
HW:TL (mg/mm)	7.79 ± 0.09*	8.10 ± 0.27^§^	6.90 ± 0.15	6.46 ± 0.13

LV, left ventricle; LVIDd/s, internal dimension diastole/systole, %EF, %ejection fraction; %FS, % fractional shortening; LVESP/DP, end systolic/diastolic pressure; LVESV/DV, end systolic/diastolic volume; SV, stroke volume; CO, cardiac output; dP/dt_max_ dP/dt_min_, derivatives of LV pressure; SBP/DBP/MAP, systolic, diastolic, mean arterial pressure; HW, heart weight; BW, body weight; TL, tibia length; *P < 0.05 *Clock*^Δ19/Δ19^-HF vs *Clock*^Δ19/Δ19^-control, ^§^*P* < 0.05 WT^-^HF vs. WT-control, ^†^*P* < 0.05 *Clock*^Δ19/Δ19^-HF vs. WT-HF by two way ANOVA followed by Tukey post hoc. Values are mean±SEM.

### Apical dendrite complexity in *Clock*^*∆19/∆19*^ versus WT mice, baseline and HF

**Figure 2:**
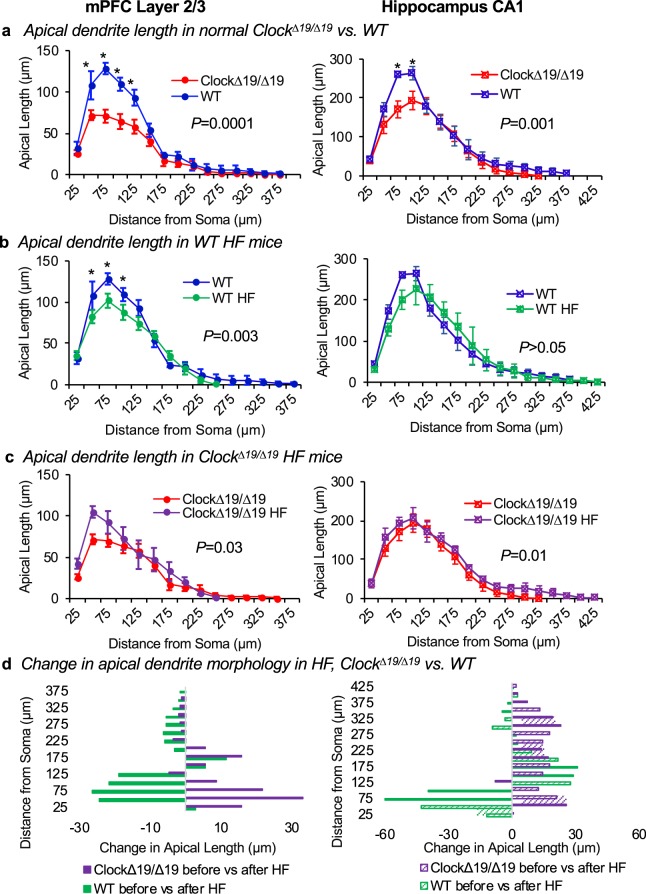
Apical dendrite morphology differs in *Clock*^*∆19/∆19*^ vs. WT mice, baseline and HF. (**a**) Apical dendrite morphology was analyzed for mPFC layer 2/3 and hippocampus CA1 pyramidal neurons using a modified three-dimensional Sholl analysis. This analysis measured the length of apical dendrite between concentric spheres radiating outward from the soma. (**a**) Normal *Clock*^*∆19/∆19*^ mice have less apical dendrite length compared to WT mice in the mPFC (left, *P* = 0.0001) and the hippocampus (right, *P* = 0.001). (**b**) WT mice with HF exhibit decreased apical dendrite length in the mPFC (left, *P* = 0.003), but no change in apical dendrite length in hippocampus (right), as compared to non-HF WT controls. In contrast, (**c**) C*lock*^*∆19/∆19*^ mice with HF exhibit increased apical dendrite length in the mPFC (left, *P* = 0.03), and increased apical dendrite length in the hippocampus (right, *P* = 0.01), versus *Clock*^*∆19/∆19*^ controls. (**d**) Thus HF is associated with changes in apical dendrite length, and the direction and magnitude of change is different in *Clock*^*∆19/∆19*^ HF versus WT HF mice, in mPFC (left) and hippocampus (right) neurons. For mPFC: n = 4 mice per baseline group, n = 5 mice per HF group. For hippocampus: n = 4 mice per baseline group, n = 4 mice per HF group. Four neurons were traced and averaged for each mouse, and data are shown as mean ± SEM. *Indicates *P* < 0.05 by Bonferroni *post-hoc* analysis.

We assessed dendrite morphology for pyramidal neurons within two regions of the brain’s cognitive system: layer 2/3 of the mPFC, and the CA1 region of the hippocampus. The size of the dendrite tree was assessed for each traced neuron using a modified three-dimensional Sholl analysis, in which the total length of dendrite matter contained between concentric spheres radiating from the soma was measured (Fig. 1c). We first assessed neurons for normal *Clock*^*∆19/∆19*^ and WT mice under baseline conditions, representative traces are shown in Fig. 1d. Remarkably, we found that *Clock*^*∆19/∆19*^ mice have mPFC neurons with less apical dendrite length, as compared to WT mice (two-way ANOVA, *F*(1, 90) = 39.7, *P* = 0.0001) (Fig. 2a left). This genotype effect is most spronounced 50 µm to 125 µm away from the soma (Bonferroni’s *post-hoc* analysis, each *P* ≤ 0.003). There is also a significant effect of distance from the soma on apical dendrite length (two-way ANOVA, *F*(14, 90) = 64.7, *P* ≤ 0.0001). Moreover, we observed differences in the hippocampus CA1 neurons, where there is less apical dendrite length in *Clock*^*∆19/∆19*^ versus WT mice (two-way ANOVA, *F*(1, 102) = 11.9, *P* = 0.001) (Fig. 2a, right). This genotype effect is most pronounced 75 µm to 100 µm away from the soma (Bonferroni’s post-hoc analysis, each *P* ≤ 0.05). There is also a significant effect of distance from the soma on apical dendrite length in hippocampus CA1 neurons (two-way ANOVA, *F*(16, 102) = 62.24, *P* ≤ 0.0001). Thus collectively, these data support our hypothesis that the circadian mechanism contributes to the neurobiology of the brain’s cognitive system, as the *Clock*^*∆19/∆19*^ mutation affects the morphology of apical dendrite trees within the mPFC and hippocampus.

We next determined whether HF is associated with changes in the neuron morphology of pyramidal neurons within the mPFC and hippocampus of WT mice. We found that apical dendrite length is lower in HF than at baseline for mPFC neurons (two-way ANOVA, *F*(1, 105) = 9.2, *P* = 0.003) (Fig. 2b, left), with no significant interaction between effects of HF and distance from the soma (two-way ANOVA, *F*(14,105) = 1.6, *P* = 0.1). In contrast, apical dendrite length is not different in HF than at baseline for CA1 neurons (two-way ANOVA, *F*(1, 102) = 0.2, *P* = 0.7) (Fig. 2b, right). Thus these data show that in WT mice, HF is associated with smaller apical dendrite trees in the mPFC layer 2/3 neurons but not in hippocampus CA1 neurons.

Next, we determined what happens in *Clock*^*∆19/∆19*^ mice with HF. Intriguingly, apical dendrite length is greater in HF than at baseline in mPFC neurons (two-way ANOVA, *F*(1, 105) = 4.6, *P* = 0.03) (Fig. 2c, left), with no interaction between the effects of HF and distance from the soma (two-way ANOVA, *F*(14, 105) = 1.0, *P* = 0.4). Apical dendrite length is also greater in HF than at baseline in hippocampus CA1 neurons (two-way ANOVA, *F*(1, 102) = 6.5, *P* = 0.01) (Fig. 2c, right), with no interaction between the effects of HF and distance from soma (two-way ANOVA, *F*(16, 102) = 0.2, *P* = 1.0). Thus these data show that in *Clock*^*∆19/∆19*^ mice, HF leads to larger apical dendrite trees in both the mPFC layer 2/3 neurons and in hippocampus CA1 neurons, and that this response is different than what happens in HF WT mice.

Finally, we compared the direction of change in apical dendrite morphology in *Clock*^*∆19/∆19*^ versus WT mice in HF. We found that the *Clock*^*∆19/∆19*^ HF mice exhibit an increase in mPFC apical dendrite length, especially in the first 100 μm from the soma, whereas the WT HF mice exhibit a large decrease in mPFC apical dendrite length near the soma (Fig. 2d, left). Intriguingly, despite the significant difference in tree size between the genotypes at baseline (Fig. 2a), there is convergence to similar end lengths across genotypes in HF (two-way ANOVA, *F*(1, 120) = 0.4, *P* = 0.5). These effects were also observed in the CA1 neurons of the hippocampus in the HF mice, as *Clock*^*∆19/∆19*^ HF mice exhibit an increase in apical dendrite length at all distances from the soma, whereas WT mice exhibit a decrease in apical dendrite length within 125 μm of the soma, and increases at further distances (Fig. 2d, right). As with the mPFC neurons, these differing directions of change in dendrite length in the CA1 hippocampus neurons leads to a convergence of similar-sized apical dendrite trees in each genotype with HF (two-way ANOVA, *F*(1, 102) = 0.1, *P* = 0.8). Thus collectively these data show that not only does neuron morphology exhibit changes with HF, but also the circadian mechanism plays a role as *Clock*^*∆19/∆19*^ HF mice exhibit differently evolving neuron morphology as compared to WT HF mice.

### Basal dendrite complexity in *Clock*^*∆19/∆19*^ and WT mice, baseline and HF

**Figure 3:**
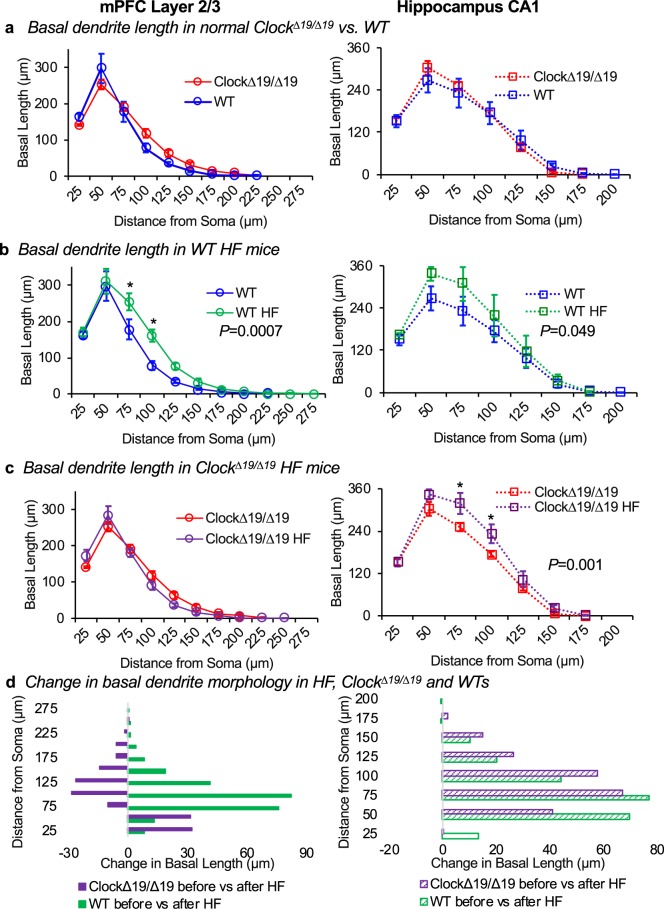
Basal dendrite morphology, baseline and HF. Basal dendrite morphology was analyzed for mPFC layer 2/3 and hippocampus CA1 pyramidal neurons using a modified three-dimensional Sholl analysis. This analysis measured the length of basal dendrite between concentric spheres radiating outward from the soma. (**a**) Normal *Clock*^*∆19/∆19*^ mice exhibit similar basal dendrite length compared to WT mice in the mPFC (left) and the hippocampus (right). (**b**) WT HF mice exhibit increased basal dendrite length in the mPFC (left, P = 0.0007), and in the hippocampus CA1neurons (right, P = 0.049), versus WT controls. In contrast, (**c**) *Clock*^*∆19/∆19*^ HF mice exhibit no difference in basal dendrite length in the mPFC (left) and increased basal dendrite length in the hippocampus neurons (right, P = 0.001), versus *Clock*^*∆19/∆19*^ controls. (**d**) Thus HF is associated with changes to basal dendrite length that differ in magnitude and direction for mPFC neurons, but are similar for hippocampus neurons, between *Clock*^*∆19/∆19*^ HF and WT HF mice. For mPFC: n = 4 mice per baseline group, n = 5 mice per HF group. For hippocampus: n = 4 mice per baseline group, n = 4 mice per HF group. Four neurons were traced and averaged for each mouse, and data are shown as mean ± SEM. *Indicates *P* < 0.05 by Bonferroni *post-hoc* analysis.

Since basal dendrite length also contributes to pyramidal neuron morphology and function, this was next assessed in *Clock*^*∆19/∆19*^ and WT mice (Fig. 3). Basal dendrite length is affected by distance from the soma in both the mPFC layer 2/3 neurons and hippocampus CA1 neurons (two-way ANOVA, both *P *< 0.0001). However, *Clock*^*∆19/∆19*^ versus WT mice show no difference in basal dendrite length, in either the mPFC (two-way ANOVA, *F*(1, 66) = 0.7, *P* = 0.4) (Fig. 3a, left) or the hippocampus (two-way ANOVA, *F*(1, 48) = 0.4, *P* = 0.8) (Fig. 3a, right). Thus, in contrast to the differences between genotypes in the apical dendrite morphology, *Clock*^*∆19/∆19*^ has no effect on the morphology of the basal dendrite trees in the mPFC layer 2/3 or in hippocampus CA1.

We then compared whether HF is associated with changes in basal dendrite length in WT mice. We found that basal dendrite length increases in HF in both mPFC neurons (two-way ANOVA, *F*(1, 77) = 12.4, *P* = 0.0007) (Fig. 3b, left) and hippocampus neurons (two-way ANOVA, *F*(1, 48) = 4.0, *P* = 0.049, Fig. 3b, right). In the mPFC neurons, this effect of HF is most pronounced at 75 µm and 100 µm away from the soma of the neuron (Bonferroni’s *post-hoc* analysis, each *P *< 0.008), but there is no interaction between the effects of HF and distance from soma in either neuron cell type (two-way ANOVA, mPFC: *F*(10, 77) = 2.0, *P* = 0.05; hippocampus: *F*(7, 48) = 0.6, *P* = 0.8). Thus these data reveal that in WT mice, there are changes in basal dendrite length associated with HF.

Next, we investigated basal dendrite length in *Clock*^*∆19/∆19*^ mice with HF. We found no change in basal dendrite length in *Clock*^*∆19/∆19*^ mice in the mPFC (two-way ANOVA, *F*(1, 77) = 0.4, *P* = 0.5) (Fig. 3c, left). Basal dendrite length in the hippocampus, however, increases (two-way ANOVA, *F*(1, 48) = 13.5, *P* = 0.001) (Fig. 3c, right). The effect of HF is most pronounced at 75 µm and 100 µm away from the soma of the neuron (Bonferroni’s *post-hoc* analysis, each P < 0.05), but there is no interaction between the effects of HF and distance from soma (two-way ANOVA, F(7, 48) = 1.8, P = 0.12). Thus collectively these data show that HF increases the size of basal dendrite trees in mPFC and hippocampus for WT mice, but only in the hippocampus for *Clock*^*∆19/∆19*^ HF mice.

Finally, we compared the direction of change in basal dendrite morphology in *Clock*^*∆19/∆19*^ HF versus WT HF mice. As with the apical dendrites, the basal dendrites also exhibit differences by genotype. In contrast to the *Clock*^*∆19/∆19*^ HF mice, the WT HF mice exhibit an increase in mPFC basal dendrite complexity (Fig. 3d, left). No difference in direction was observed between genotypes in response to HF in basal dendrites of hippocampal neurons (Fig. 3d, right). Thus, these data further support the evolution of neuron morphology in HF, and that the circadian mechanism plays a role, as mutation of CLOCK alters the morphological response in basal dendrites differently as compared to the WT HF response.

### Visual-spatial memory differs in *Clock*^*∆19/∆19*^ and WT mice

**Figure 4:**
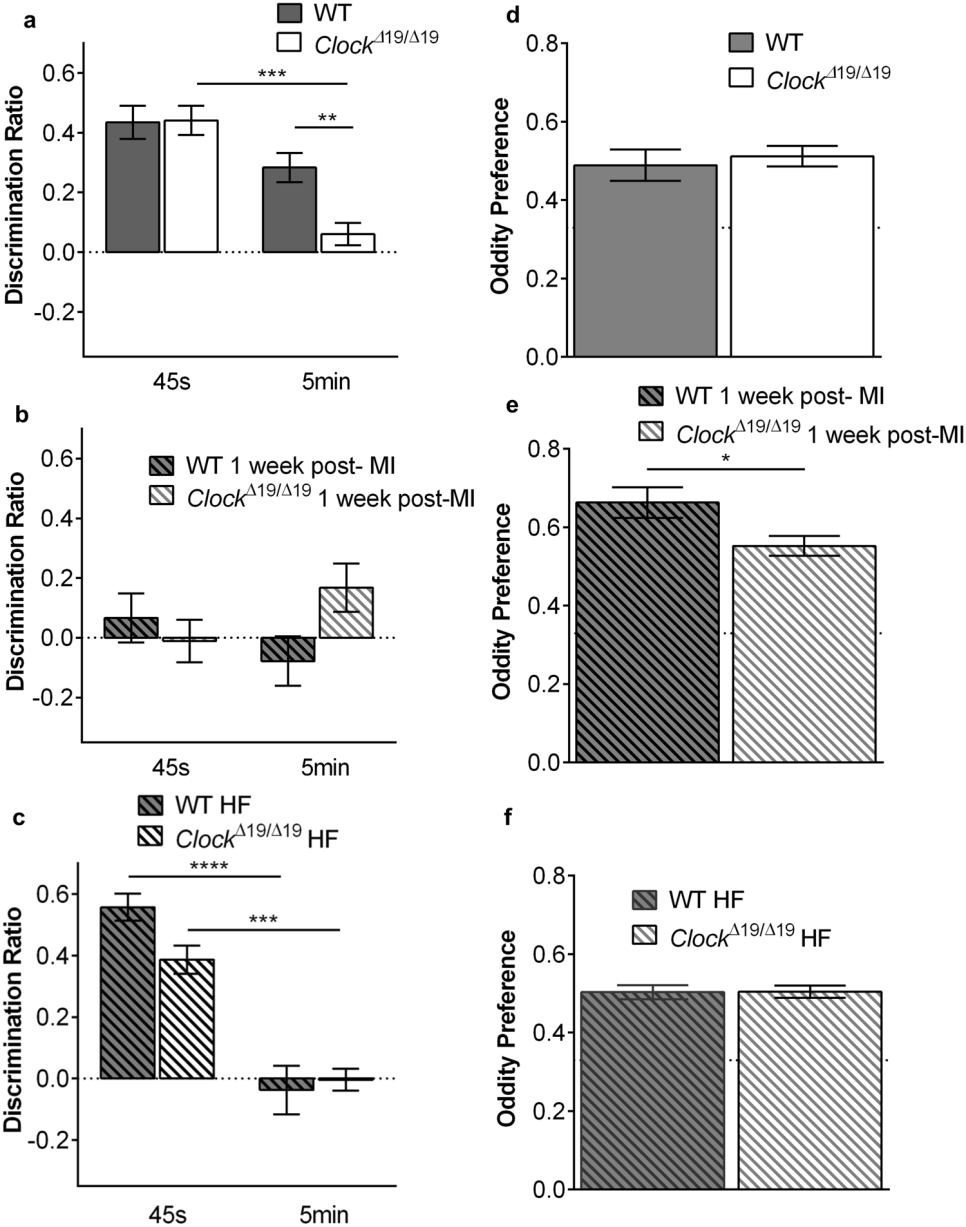
Visual-spatial memory differs in *Clock*^*∆19/∆19*^ and WT mice. (**a**) At baseline, *Clock*^*∆19/∆19*^ mice show impaired short term OiP memory (5 minutes) compared to WTs (n = 23 *Clock*^*∆19/∆19*^, n = 27 WT). (**b**) At 1 week post-myocardial infarction, OiP performance is impaired in both *Clock*^*∆19/∆*19^ mice and WT mice at both immediate (45 seconds) and 5-minute retention delays (n = 16 *Clock*^*∆19/∆19*^, n = 17 WT). (**c**) In the 8-week HF mice, OiP performance is impaired in *Clock*^*∆19/∆*19^ vs. WT mice at immediate (45 second) delays, and is for both *Clock*^*∆19/∆19*^ and WT mice at 5-minute retention delays (n = 16 *Clock*^*∆19/∆19*^, n = 17 WT). Object oddity discrimination is similar at (**d**) baseline (n = 5/group), (**e**) 1 week post-myocardial infarction (n = 6/group), and in the (**f**) 8 week HF mice (n = 16 WT, n = 17 *Clock*^*∆19/∆19*^), as the oddity preference significantly differed from 0.33 (chance performance). However, *Clock*^*∆19/∆19*^ mice performed worse than WT at 1 week post-myocardial infarction *P < 0.05, **P < 0.01, ***P < 0.001, ****P < 0.0001.

We next assessed whether there were possible functional consequences of the differing neuron morphology in *Clock*^*∆19/∆19*^ versus WT mice. A mPFC- and hippocampus-dependent object in place (OiP) memory task was first run on the mice. At baseline, *Clock*^*∆19/∆19*^ mice show impaired short term (5 minutes) OiP memory compared with WT mice (two-way split-plot ANOVA: genotype F(1, 48) = 4.87, *P* < 0.001, delay F(1, 48) = 28.72, *P* < 0.05, and genotype × delay F(1, 48) = 5.33, *P* < 0.05; WT n = 27, *Clock*^*∆19/∆19*^ *n* = 23). Compared with WT mice, *Clock*^*∆19/∆19*^ mice exhibit a lower discrimination ratio at the 5 minute retention delay (t(48) = 3.52, *P *< 0.01) (Fig. 4a). *Clock*^*∆19/∆19*^ mice also perform worse at the 5 minute delay than the immediate delay (t(22) = 5.94, *P *< 0.001). One-sample t-tests suggest intact memory in WT mice (45 seconds: t(26) = 7.78, P < 0.001; 5 minutes: t(26) = 5.80, *P* < 0.001), and *Clock*^*∆19/∆19*^ mice at 45 seconds (t(22) = 8.94, *P* < 0.001), but impaired memory in *Clock*^*∆19/∆19*^ mice at 5 minutes (t(22) = 1.60, *P* = ns), as the discrimination ratio did not differ from zero (chance performance). Thus these data indicate that CLOCK may have functional consequences leading to differences in OiP memory tasks.

We then compared what happens in *Clock*^*∆19/∆19*^ and WT mice with HF. The first time point of 1 week was first investigated as it is a robust period of cardiac remodeling including inflammatory processes such as elaboration of cytokines which can influence cognitive function. We found that OiP memory is impaired in both WT and *Clock*^*∆19/∆19*^ mice, at both retention delays (two-way split-plot ANOVA: genotype x delay F(1, 31) = 4.60, *P *< 0.05, WT n = 17, *Clock*^*∆19/∆19*^) (Fig. 4b). One-sample t-tests suggest impaired memory in all conditions (WT 45 seconds: t(16) = 0.81, *P* = ns; WT 5 minutes: t(16) = 0.95, *P* = ns; *Clock*^*∆19/∆19*^ 45 seconds: t(15) = 0.16, *P* = ns; *Clock*^*∆19/∆19*^ 5 minutes: t(15) = 2.08, *P* = ns, as the discrimination ratios did not differ from zero. Thus, myocardial infarction is associated with impaired OiP memory tests for both genotypes during the early remodeling period.

Next, we investigated what happens in HF by 8 weeks after myocardial infarction, by which time the observed changes in neuron morphology had occurred (e.g. Figs 2d and 3d). In our HF mice, we found that OiP memory is impaired at 5 minutes in both WTs and *Clock*^*∆19/∆19*^ (two-way split-plot ANOVA: delay F(1, 31) = 58.46, *P *< 0.001, WT n = 17, *Clock*^*∆19/∆19*^ *n* = 16) (Fig. 4c). One-sample t-tests suggest intact memory at 45 seconds (WT: t(16) = 12.58, *P *< 0.001; *Clock*^*∆19/∆19*^: t(15) = 8.49, *P *< 0.001), but impaired memory at 5 minutes (WT: t(16) = 0.47, *P* = ns; *Clock*^*∆19/∆19*^: t(15) = 0.91, *P* = ns), as the discrimination ratio did not differ from zero. Taken together, these data suggest that as HF pathophysiology progresses, it is associated with ongoing impairment in OiP memory tests.

In contrast to the data with OiP memory tests, object oddity discrimination in HF remains intact across all time points (Fig. 4d–f). One-sample t-tests suggest intact object oddity discrimination across all time points (baseline WT: t(4) = 3.92, *P *< 0.05; baseline *Clock*^*∆19/∆19*^: t(4) = 6.94, *P *< 0.01; 1 week post-myocardial infarction WT: t(5) = 8.49, *P *< 0.001; 1 week post-myocardial infarction *Clock*^*∆19/∆19*^: t(5) = 8.72, *P *< 0.001; 8 weeks HF WT t(16) = 9.64, *P *< 0.001; 8 weeks HF *Clock*^*∆19/∆19*^: t(15) = 11.30, *P *< 0.001), as the oddity preference significantly differs from 0.33 (chance performance). However, *Clock*^*∆19/∆19*^ mice perform worse than WT at 1 week post-infarction (t(10) = 2.36, *P *< 0.05) (Fig. 4e).

### Circadian regulation of cerebrovasculature

**Figure 5:**
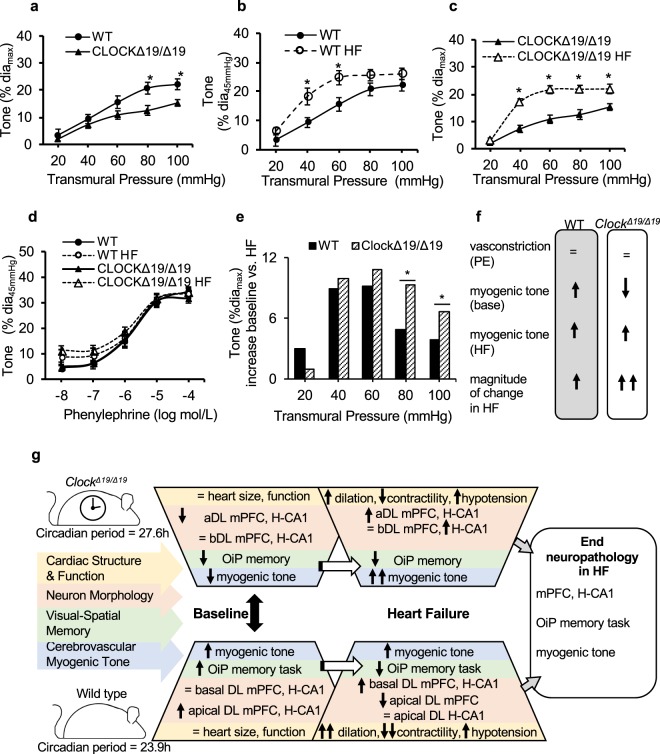
Pressure myography of posterior cerebral arteries, neurobiology of HF. (**a**) At baseline, *Clock*^*∆19/∆19*^ PCA have lower myogenic tone versus WT PCA at all transmural pressures >60 mmHg. In the HF mice, and compared to baseline, (**b**) WT PCA exhibit increased myogenic tone at transmural pressures of 40 mmHg and 60 mmHg, whereas (**c**) *Clock*^*∆19/∆19*^ PCA have increased myogenic tone at all transmural pressures tested>20mmHg. (**d**) PCA of all groups respond similarly to phenylephrine, suggesting similar capability to respond. However, as compared to WT PCA, the *Clock*^*∆19/∆19*^ PCA exhibit reduced myogenic tone at baseline, and (**e**) a greater delta change in myogenic tone in response to HF (**e**), supporting the notion that the circadian mechanism can influence responses in cerebrovasculature. (**f**) Summary of the different responses of WT PCA versus *Clock*^*∆19/∆19*^ PCA. All PCA were collected during the middle of the animals’ wake period (Zeitgeber time (ZT19)). *P < 0.05, n = 6 WT PCA, n = 8 WT HF PCA, n = 7 *Clock*^*∆19/∆19*^ PCA, n = 7 *Clock*^*∆19/∆19*^ HF PCA. (**g**) The circadian mechanism is an important regulator of healthy cognitive system neurobiology. Neurobiological adaptations to HF differ in magnitude and direction in *Clock*^*∆19/∆19*^ versus WT mice, including neuron morphology, visual-spatial memory and cerebrovascular myogenic tone, leading to convergence of end stage measures. These findings highlight the need to better understand how the circadian mechanism affects neurobiological adaptations to HF, a leading cause of morbidity and mortality worldwide. Large black double-sided arrow denotes comparison of normal *Clock*^*∆19/∆19*^ and WT mice. Open white single sided arrows denote comparison of each genotype at baseline and in HF.

Neurological deficits in HF may correlate with altered blood flow regulation in small vessels, thus we next performed pressure myography to characterize the tone in the posterior cerebral arteries (PCA) of *Clock*^*∆19/∆19*^ and WT mice. At baseline, the myogenic responsiveness in *Clock*^*∆19/∆19*^ PCA is significantly lower than in WTs (*P *< 0.05 at pressures>60 mm Hg) (Fig. 5a). We then compared whether HF leads to changes in myogenic tone in WT mice. We found that the myogenic responsiveness is significantly increased in the HF WT PCA at 8 weeks post-myocardial infarction, as compared to controls (P < 0.05 at pressures of 40 mmHg and 60 mmHg) (Fig. 5b). Next, we investigated myogenic tone in *Clock*^*∆19/∆19*^ PCA in HF. We found that the myogenic responsiveness is also significantly increased in the HF *Clock*^*∆19/∆19*^ PCA by 8 weeks post-myocardial infarction versus controls, but moreover this increase in tone occurs over broader range of physiologic pressures and is greater in magnitude than WT PCA (P < 0.05 at pressures>20 mmHg to 100 mmHg) (Fig. 5c). Intriguingly, PCA of all 4 groups show similar vasoconstriction to phenylephrine and thus similar capacity (Fig. 5d), however, *Clock*^*∆19/∆19*^ PCA exhibit lower myogenic tone at baseline, and a greater rise in myogenic tone in HF, as compared to WT PCA (Fig. 5e). These differences in cerebrovascular tone in *Clock*^*∆19/∆19*^ versus WT PCA, and in HF, are illustrated in Fig. 5f. Circadian regulation of healthy cognitive systems neurobiology, and adaptations to HF are summarized in Fig. 5g.

### Neural gene expression differs in response to heart disease

**Figure 6:**
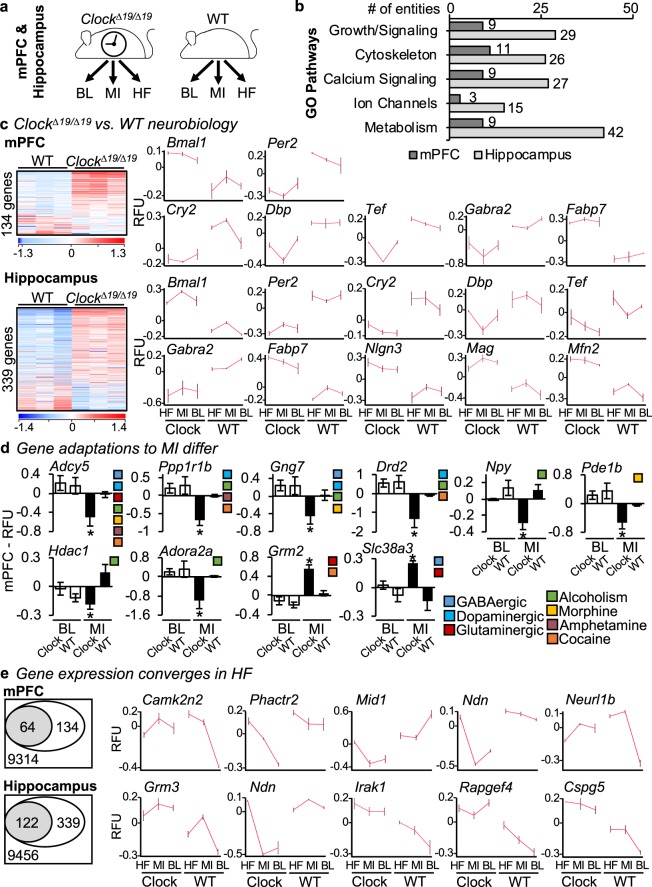
Gene expression in *Clock*^*∆19/∆19*^ versus WT mPFC and hippocampus, and adaptations in response to HF. (**a**) Gene expression was assessed in *Clock*^*Δ19/Δ19*^ and WT mPFC and hippocampus at baseline (BL), after MI, and in HF, by using microarrays and bioinformatics analyses. (**b**) Key Gene Ontology (GO) pathways identified for genes with ≥ 1.3-fold difference in *Clock*^*Δ19/Δ19*^ versus WT mice, in the mPFC (dark boxes) or hippocampus (light boxes). (**c**) Baseline conditions. Circadian mechanism and output genes that differ in expression (≥ 1.3-fold change) in the mPFC (top) or hippocampus (bottom) for *Clock*^*Δ19/Δ19*^ versus WT mice. (**d**) Response to MI. Genes that differ in expression (≥ 1.3-fold change) in the mPFC of *Clock*^*Δ19/Δ19*^ versus WT mice. (**e**) Response to HF. *Clock*^*Δ19/Δ19*^ versus WT genes that exhibit dichotomous expression at baseline, and then converge in HF, mPFC (top) or hippocampus (bottom). For images in Fig. 6, RFU  =  relative fluorescence units, *P < 0.05 by Student’s *t*-test, further information on genes shown is provided in Table 2, and all gene shown as well as additional identified genes are further detailed in Supplementary Table S1.

Finally, we investigated mechanisms underlying the dichotomous neurobiological responses between *Clock*^*∆19/∆19*^ and WT mice. We found no obvious differences in early cardiac remodeling - inflammasome genes and serum cytokines post-MI (Supplementary Fig. S1). These findings are consistent with the notion that rather than being systemically driven, CLOCK regulated neurobiological responses occur at a cellular level in the brain. Thus we next examined gene expression in the mPFC and hippocampus, using microarrays and bioinformatics analyses. Global gene profiles were determined at baseline (BL), MI, and HF, in the mPFC and hippocampus of *Clock*^*∆19/∆19*^ and WT mice (Fig. 6a). We found significant differences in genes involved in neural growth, cytoskeleton, signalling, and metabolism in the *Clock*^*∆19/∆19*^ mice (Fig. 6b), consistent with the notion that CLOCK acts at a cellular level. The genes plotted are defined in Table 2, and these and additional genes are further detailed in Supplementary Table S1.

**Table 2:** Key genes underlying dichotomous neurobiological responses in *Clock*^*∆19/∆19*^ mice.

Gene	Gene Name
*Bmal1*	Brain And Muscle ARNT-Like 1
*Per2*	Period 2
*Cry2*	Cryptochrome 2
*Dbp*	D Site Of Albumin Promoter (Albumin D-Box) Binding Protein
*Tef*	Thyrotrophic Embryonic Factor
*Gabra2*	Gamma-Aminobutyric Acid Type A Receptor Alpha2 Subunit
*Fabp7*	Fatty Acid Binding Protein 7
*Adcy5*	Adenylate Cyclase 5
*Ppp1r1b*	Protein Phosphatase 1 Regulatory Inhibitor Subunit 1B
*Gng7*	G Protein Subunit Gamma 7
*Drd2*	Dopamine Receptor D2
*Npy*	Neuropeptide Y
*Pde1b*	Phosphodiesterase 1B
*Hdac1*	Histone Deacetylase 1
*Adora2a*	Adenosine A2a Receptor
*Grm2*	Glutamate Metabotropic Receptor 2
*Slc38a3*	Solute Carrier Family 38 Member 3
*Camk2n2*	Calcium/Calmodulin Dependent Protein Kinase II Inhibitor 2
*Phactr2*	Phosphatase And Actin Regulator 2
*Mid1*	Midline 1
*Ndn*	Necdin
*Neurl1b*	Neuralized E3 Ubiquitin Protein Ligase 1B
*Nlgn3*	Neuroligin 3
*Mag*	Myelin Associated Glycoprotein
*Mfn2*	Mitofusin 2
*Grm3*	Glutamate Metabotropic Receptor 3
*Ndn*	Necdin
*Irak1*	Interleukin 1 Receptor Associated Kinase 1
*Rapgef4*	Rap Guanine Nucleotide Exchange Factor 4
*Cspg5*	Chondroitin Sulfate Proteoglycan 5

Genes listed are as profiled in Fig. 6, and were identified as significantly different in the mPFC, or hippocampus, or both. Further details about these genes are provided in Supplementary Table S1, including Affymetrix identifiers, fold change values, gene expression values under all conditions tested (baseline, MI, HF) in *Clock*^*∆19/∆19*^ and WT mice, chromosome location, and tags to esemble, entrezgene, genebank and GO databases. Additional genes identified but not mapped in Fig. 6 are also provided in Supplementary Table S1.

We investigated three paradigms. First, we determined which genes differed at baseline in the mPFC and/or hippocampus of healthy *Clock*^*∆19/∆19*^ mice. We found that the core circadian mechanism and its output genes were remarkably dysregulated in the *Clock*^*∆19/∆19*^ mPFC, and hippocampus, consistent with the notion that the circadian mechanism contributes to neurobiological adaptations within cognition-relevant brain regions (Fig. 6c). Second, we investigated neurobiological gene adaptations to MI. In the mPFC, we found significant differences in genes involved in neuron signalling, and in stress (addiction) pathways in the *Clock*^*∆19/∆19*^ mice (Fig. 6d). In the hippocampus, differences predominantly mapped to metabolic pathways (data not shown, lists are in Supplementary Table S1). Finally, we investigated why *Clock*^*∆19/∆19*^ mice respond differently to HF, versus WT mice. To do this, we profiled stress response pathways in the brain, with a focus on altered stress response signalling at baseline in *Clock*^*∆19/∆19*^ mice, and how these same genes responded during HF where there is phenotypic convergence (Fig. 6e). We identified unique cassettes of 64 genes in the mPFC (top) and 122 genes in the hippocampus (bottom) in which altered expression at baseline converged in response to HF. Taken together, these findings support the notion that the circadian mechanism influences normal neurobiology, and provide insights into the role of CLOCK in neurobiological adaptations within cognition-relevant brain regions in HF.

## Discussion

The circadian mechanism plays a critical role in cardiac remodeling in HF. Since neurological conditions (e.g. cognitive impairment, depression) frequently coincide in patients with HF, we hypothesized that the circadian mechanism also influences neuropathology in HF. In this study, we found that CLOCK is pivotal to maintain normal neuron morphology; *Clock*^*∆19/∆19*^ mice have smaller apical dendrite trees in mPFC layer 2/3 and hippocampus CA1 neurons, versus WT mice. We also observed functional consequences, as visual-spatial memory differs in *Clock*^*∆19/∆19*^ versus WT mice on the OiP memory tasks. Moreover, normal *Clock*^*∆19/∆19*^ mice exhibit lower cerebrovascular myogenic tone, versus WT mice. We then investigated what happens in HF, by using the left anterior descending coronary artery ligation model. We observed significant differences in the magnitude and direction of adaptations to HF, including those to neuron morphology in the mPFC and hippocampus, visual-spatial memory, and PCA myogenic responsiveness in *Clock*^*∆19/∆19*^ versus WT mice. Intriguingly, despite the differences in adaptations to HF, they converged towards similar end profiles in neuron morphology, memory, and cerebrovascular tone in *Clock*^*∆19/∆19*^ and WT mice. This dichotomous response was mediated in part by differences in baseline gene expression in growth and stress response pathways in neurocognitive regions in the brains of *Clock*^*∆19/∆19*^ mice, followed by differential activation of neural mRNA pathways in response to MI, with convergence in HF. Collectively these studies establish a new connection between the circadian mechanism and cognitive system neurobiology, and mechanisms underlying adaptations to this system in HF.

One of the novel findings of this study is that the circadian mechanism, and specifically CLOCK, influences cognitive system neurobiology. We used a genetic approach to elucidate a role for CLOCK in neuron morphology/function. We found that *Clock*^*∆19/∆19*^ mice have pyramidal neurons with altered dendrite trees within layer 2/3 of the mPFC and in CA1 of the hippocampus, and impairments in visual-spatial memory, as compared to WT mice. This is the first study showing that CLOCK plays a role in maintaining neuronal health, and adds to the growing notion that core clock genes regulate aspects of neuron cell biology. Indeed, it was previously shown in mice that loss of another circadian mechanism gene, namely *Bmal1*, induces astrogliosis in the cerebral cortex and hippocampus^38^, and is associated with impairments in learning and memory^39^. Moreover, several recent environmental studies provide indirect support for the notion that the circadian mechanism mediates neuron morphology and cognition. For example, circadian desynchrony in hamsters, induced by experimental “jet lag”, decreases hippocampal cell proliferation and neurogenesis, and impairs hippocampus-dependent learning and memory^40^. Another study showed that inducing circadian desynchrony by altering the external light and dark environment reduces the size of apical dendrite trees for pyramidal neurons in the mPFC, and impairs ability to shift learned behaviors^41^. Thus, CLOCK contributes to the neuron morphology and dependent function of neurons within cognitive circuits. These findings also provide a foundation for investigating a novel role for the circadian mechanism in the neuropathology of HF.

Another key finding is that there are changes in neuron morphology in the mPFC and hippocampus associated with HF, and that this correlates temporally with impaired cognitive responses. Recent clinical studies support our findings that HF affects the brain and contributes to cognitive and emotional abnormalities. For example, patients with HF have damage in cognitive regulatory areas including the cerebral cortex and hippocampus^42^, and emotional deficits are commonly found among patients with ischemic heart disease and HF^2–4,43–46^. Experimentally, De Sliva and colleagues recently showed that mice subjected to myocardial ischemia/reperfusion (mI/R) exhibit reactive gliosis throughout the hippocampus, and impaired performance on fear-conditioning and on object location memory tests^47^, thus providing further support for our findings. It is worth noting that here we used a model of HF induced by permanent ligation of the coronary artery, which produces larger infarcts and more rapid progression to HF than mI/R. This bears clinical relevance, as many human patients post-MI do not reach hospitals in a timely fashion and undergo reperfusion, or the procedure is unsuccessful with incomplete revascularization or no reflow.

We also investigated whether the circadian mechanism directly acted on cerebrovascular blood flow, as might be expected to regulate perfusion in HF. In support of this approach the circadian mechanism is implicated in a regulation of diverse vascular beds^48–50^, and in the regulation of daily patterns of blood pressure in health and disease (reviewed in^51^). This is regulated in large part by myogenic response, which is a mechanism employed by resistance arteries to match resistance transmural pressure, and an important regulator of blood flow^52^. We found that the *Clock*^*∆19/∆19*^ mice significantly increased their PCA in HF, as compared to normal *Clock*^*∆19/∆19*^ mice. Moreover, this response was greater in magnitude and over a wider range of physiological pressures, as compared with the WTs in HF. However, we also observed that there was no difference when we compared the overall myogenic responsiveness of *Clock*^*∆19/∆19*^ HF and WT-HF mice, suggesting that while they exhibited a different magnitude of response, the end outcomes were similar. This suggests that the mechanism may not be at the level of cerebrovascular blood flow in *Clock*^*∆19/∆19*^ HF mice, as regulation is similar in that vascular bed. That is, it seems likely that CLOCK influences adaptations at a cellular level, whether neuron or vascular cell, and not at the systems level.

To investigate how CLOCK influences neuronal adaptations at a cellular level, we asked two important questions. First, we investigated which genes differed in the cognition relevant brain regions of *Clock*^*∆19/∆19*^ versus WT mice, which could help to explain the neurobiological impairments in the normal *Clock*^*∆19/∆19*^ mice. Microarray and bioinformatics analyses revealed that *Clock*^*∆19/∆19*^ mice exhibit altered expression of genes involved in neural morphology, signaling, and metabolism. For example, one of the genes identified was *gamma-aminobutryic acid type A receptor alpha2 subunit* (*Gabra2*) gene, which plays a critical role in stress responses in the brain^53^. We also found differences in the *fatty acid binding protein 7* (*Fabp7*) gene, which is involved in neural structure in the developing brain^54,55^. Differences were also found in *neuroligin 3* (*Nlgn3*) and in *myelin associated glycoprotein* (*Mag*), which are involved in neural cell interactions^56^, and in mitofusin 2 (*Mfn2*) which is involved in mitochondrial activities^57^. These genes are highlighted in Fig. 6c, Table 2, and additional details and genes are in Supplementary Table S1. Intriguingly, gene expression appeared to be differentially expressed brains of healthy *Clock*^*∆19/∆19*^ mice, as well as under the disease conditions of MI and HF. The holding expression pattern greatly resembles what is also observed with the circadian mechanisms genes that are a part of CLOCK transcriptional control. Thus these observations suggest that these new genes may also be under CLOCK regulatory control; that is, novel output genes regulated by the circadian mechanism in the brain.

Second, we investigated how gene adaptations differed in response to heart disease in the *Clock*^*∆19/∆19*^ versus WT mice. We especially focused on genes that showed altered stress responses in signaling at baseline, but then converged and were similar in HF; these could help explain the phenotypic convergence observed at HF. The genes are highlighted in Fig. 6e, Table 2, and additional details and genes are in Supplementary Table S1. Some of the genes identified may directly trigger neuronal adaptations at the cellular level because they regulate key physiological functions such as neuronal synaptic plasticity (e.g. *calmodulin dependent protein kinase II inhibitor 2, Camk2n2*), neural growth and differentiation (e.g. *Necdin, Ndn; chondroitin sulfate proteoglycan 5, Cspg5*), and neurotransmission (e.g. *glutamate metabotropic receptor 3, Grm3*). However, these genes may also indirectly trigger neuronal adaptations at the cellular level; that is, they can make the cells more susceptible to secondary consequences. For example, changes in the neural cell membranes may increase susceptibility to damage from oxidative stress in both *Clock*^*∆19/∆19*^ and WT mice; indeed, oxidative stress mediators are well known to play an important role in the pathophysiology of HF^58^. Thus taken together, differential activation of the genes in these pathways can help to explain how CLOCK drives dichotomous neurobiology that converges in HF.

One additional point worth noting is that these studies were done in male mice. However, there has been a flurry of recent studies describing how heart disease manifests differently by biological sex and gender, and importantly, that the circadian clock mechanism plays a role^21,24,59–61^. Future studies examining differences in genes expression in male versus female brain regions are warranted, especially in the context of designing circadian medicine based therapies to reduce neurocognitive impairments in HF patients of both biological sexes.

The results of this study have important clinical implications for patients with circadian rhythm disturbances. In humans recovering from MI, circadian desynchrony can occur as a consequence of environmental conditions in our intensive and coronary care units. That is, frequent patient-staff interactions at night, light and noise conspire to disturb sleep and circadian rhythms^62,63^. Experimentally, circadian rhythm desynchrony, even short-term for just the first few days after MI, impairs healing and exacerbates maladaptive cardiac remodeling in the murine model^23^. We would postulate, based on this study, that circadian rhythm disturbances will exacerbate the neurobiological impairments that develop in MI patients. Moreover, subsequent circadian disturbances could cause further neurobiological impairments as patients progress to HF. For example, individuals are subjected to a wide variety of circadian rhythm disturbances in contemporary society such as through night shift work, sleep disorders, and social jet lag (e.g. reviewed in^18^). Studies investigating the prevalence and severity of neurobiological changes in shift workers who develop HF, or patients with sleep disorders and HF, are clearly indicated. Understanding how the circadian mechanism contributes to neuropathology can lead to new strategies to reduce neurocognitive impairment and improve the quality of life for patients with HF.

In summary, we show that the circadian mechanism influences neurobiology in the brain’s cognitive systems. The circadian mechanism is an important regulator of healthy cognitive system neurobiology, and loss of CLOCK leads to adverse changes in the neurobiology of the cognitive system. These data also elucidate a role for the circadian mechanism in neurocognitive adaptations in HF. This is important because clinically many patients with HF often present with concurrent cognitive impairments, and there is no cure for HF. New understanding of the circadian mechanism, and it’s role in brain pathophysiology, can lead to new approaches to reduce morbidity and improve the quality of life for HF patients.

## Methods

### Animals

All studies were approved by the University of Guelph Institutional Animal Care and Use Committee and in accordance with the guidelines of the Canadian Council on Animal Care. Male C57Bl/6 mice were obtained from Charles River, Quebec, Canada. Male *Clock*^*∆19/∆19*^ mice^35^ were obtained from our breeding colony at the University of Guelph, and genotyped as described previously^21–24^. All mice were housed in the Central Animal Facility at the University of Guelph under a 12-hour light (L) and 12-hour dark (D) cycle with lights on at 8:00am (Zeitgeber Time 0 (ZT0)) and lights off at 8:00 pm (ZT12), and were provided with food and water *ad libitum*. Activity was recorded from individual cages equipped with running wheels and analyzed using ClockLab (Actimetrics)^21–23^.

### Left anterior descending coronary artery ligation model and pathophysiology

Mice (8 weeks of age) were subjected to left anterior descending coronary artery ligation for 8 weeks (HF model) and pathophysiologic assessments, as described previously^23,24^. Briefly, mice were anesthetized with isoflurane, intubated, and ventilated (Harvard Apparatus Model 687) throughout the procedure. A local anesthetic of 50:50 bupivacaine and lidocaine solution was administered prior to incision. An incision was made at the 3^rd^ intercostal space on the left side. A prolene 7-0 suture (Ethicon) was passed underneath the LAD at 1mm below the edge of the left auricle, and ligated. The chest and skin were closed with silk 6-0 sutures (Ethicon). All surgeries were performed in a very short window of time, between ZT01 and ZT03. Shams were subjected to the same procedures, but without LAD coronary artery ligation. Mice were administered buprenorphine (0.1 mg/kg) upon awakening.

Pathophysiologic assessments were made prior to procedures, and at 1 week post-myocardial infarction, and at 8 weeks post-infarction (HF model), by echocardiography and *in-vivo* hemodynamics analyses, as described previously^21–24,26,60^. Briefly, cardiac function and morphometry were assessed in a blinded manner under light anesthesia (1.5% isoflurane), on a GE Vivid7 Dimension ultrasound equipped with a 14 MHz linear-array transducer. All measurements were acquired at the mid-papillary level, to determine LV internal dimensions at end-diastole (LVIDd), LV internal dimensions at end-systole (LVIDs), % ejection fraction (EF), % fractional shortening (FS) and heart rate (HR). At least 5 different images were analyzed per heart, with n = 8 hearts per group. For *in vivo* hemodynamics, mice were placed under 3.5% isoflurane, intubated, and body temperature was continuously monitored and maintained at 37 °C. A 1.2-Fr pressure-volume catheter (Transonic) was advanced into the LV, and measurements were recorded using an ADInstrument PowerLab. LV end systolic pressure (LVESP) and end diastolic pressure (LVEDP), and volumes (LVESV, LVEDV), stroke volume (SV), cardiac output (CO), maximum and minimum first derivative of LV pressure (dP/dtmax, dP/dtmin), and systolic and diastolic blood pressure (SBP, DBP) were recorded. Mean arterial blood pressure (MAP) was calculated as DBP+[(SBP−DBP)/3]. Following collection of hemodynamics data, mice were sacrificed by isoflurane overdose and cervical dislocation. Body weight (BW), heart weight (HW) and tibia length (TL) measurements were collected.

### Brain collection and Golgi-Cox staining

HF mice were sacrificed at 8 weeks post-myocardial infarction (and age-matched controls) by isoflurane and cervical dislocation. Mice were decapitated and the brain quickly removed. Golgi-Cox staining was performed^64,65^. Briefly, whole brains were immediately placed into Golgi-Cox impregnation solution (1% potassium dichromate, 0.8% potassium chromate, 1% mercuric chloride) and incubated in this solution for 25 days in the dark at room temperature. Brain sections containing the mPFC or dorsal hippocampus were made at 500 µm thickness using a vibratome, developed using ammonium hydroxide, fixed with Kodak Fixative A, mounted onto microscope slides, dehydrated in ethanol gradients, then cover slipped using anhydrous mounting medium.

### Neuron imaging, tracing, and morphology analysis

Neuron imaging and tracing were performed^64^. Briefly, neurons were imaged in bright field using an Olympus BX53 upright microscope (Olympus, Richmond Hill, ON, Canada) controlled using Neurolucida software (version 10, MBF Bioscience, Williston, VT). Overlapping image stacks containing neurons to be traced were captured using an Olympus UPlanSApo 30X, 1.05 NA silicon oil immersion objective. Pyramidal neurons in layer 2/3 of the mPFC and the CA1 region of the hippocampus were imaged and manually traced in three dimensions using the Neurolucida software. All experimenters were blinded to the treatment group prior to neuron imaging and tracing. Four neurons were sampled randomly from each brain region, from n = 4–5 mice per group, based on the criteria of: (i) being fully contained within the thickness of the slice, (ii) not being occluded by other stained neurons, and (iii) having stained dendrites that were fully intact. Quantitative analyses of apical and basal dendrites for each neuron were performed using Neurolucida Explorer (MBF Bioscience). Data for the neurons within each brain region were averaged per mouse, and statistical analyses were performed with the mouse serving as the level of sample.

### Object-in-place (OiP) memory task

Four objects varying in size (7–20 cm), color, material (glass, aluminum, ceramic and plastic) and texture were placed in each corner of an open field, 5 cm away from the walls as described previously^66^. The objects were washed with 50% ethanol between trials to eliminate exploration bias due to olfactory cues. The open field (45 × 45 × 30 cm^3^) contains no spatial cues on the apparatus walls and a bare floor. It was constructed of white, plastic-coated corrugated cardboard. Spatial cues were present in the testing room (i.e. television, shelving, camera and colored door), and a ceiling-mounted white light illuminated the room. All mice experienced two habituation sessions on successive days, where they explored an empty open field for 10 min. Learning occurred 1 day following habituation, during a 10-minute sample phase where mice were allowed to freely explore four different novel objects. To assess immediate and short term memory, mice experienced a 2-minute choice phase 45 seconds and 5 minutes later where they viewed the same four objects, but two objects had switched locations (right or left, counterbalanced) creating a ‘novel side’. The discrimination ratio (DR) was calculated as [(novel object exploration − familiar object exploration)/(total object exploration)]. Preference for the objects on the novel side gave a DR value significantly greater than zero, and was interpreted as being indicative of memory. MI surgeries on all mice occurred 1 day following the last choice phase. At 1 week and 8 weeks following MI surgeries, the mice repeated the same sample and choice phases as previously described with an additional set of 4 novel objects for each time point. Mouse exploration was scored by two researchers. 95% interrater reliability was maintained to ensure consistency in the recording of data.

### Object oddity perceptual task

To assess whether there was impairment of basic object perceptual discrimination following MI, an object oddity task was used after OiP testing, as described previously^67,68^. The same open field used for the OiP test was used for this task. Three objects, two identical, one unique, were place on one side of the open field, 5 cm away from the wall. Mice experienced a single 10-minute sample phase in which they were allowed to freely explore the three objects. To ensure object preference was due to one object being unique rather than a bias for an object, the order of the three objects and the selection of the unique object were switched after each mouse. Oddity preference was calculated as [unique object exploration]/[total exploration]. An oddity preference ≥0.33 indicated a greater preference for the unique object and intact perception. As with the OiP task, mouse exploration was scored by two researchers. 95% interrater reliability was maintained to ensure consistency in the recording of data.

### Pressure myography

Mouse PCA were isolated, cannulated, and underwent pressure myography as previously described^69,70^. All PCA were collected during the middle of the animals’ wake period (Zeitgeber time (ZT19)). Briefly, PCA were isolated in 4 °C MOPS-buffered salt solution containing (mmol/L): NaCl 145, KCl 4.7, CaCl_2_•2H_2_O 1.5, MgSO_4_•7H_2_O 1.17, NaH_2_PO_4_•2H_2_O 1.2, pyruvate 2.0, EDTA 0.02, MOPS (3-morpholinopropanesulfonic acid) 3.0, and glucose 5.0. Arteries were cannulated in a pressure myograph (Living Systems Instruments, Vermont) on glass micropipettes and a servo-controlled pump provided the inflow pressure (Living Systems). Outflow pressure was blocked providing a no-flow condition. Diameter was imaged using an inverted microscope (Nikon TMS) and CCTV camera (Panasonic) and the image was relayed through edge detection software (Dataq DS-720) to a personal computer. Vessels were set to *in vivo* pressures (45 mmHg) and gradually heated to 37 °C. Only vessels that vasoconstricted robustly (>30%) to the α_1_-agonist L-phenylephrine (PE, 100 μmol/L, Sigma-Aldrich) were considered viable and underwent the subsequent protocol. Vasoconstriction was assessed in response to a range of PE concentrations (10 nmol/L– 100 μmol/L). Pressure-induced myogenic responsiveness was assessed by step-wise increases in transmural pressures (20 to 100 mmHg in 20 mmHg increments, 5 min at each). Arteries were then incubated in Ca^2+^-free MOPS-buffered saline solution ([mmol/L]: NaCl 147, KCl 4.7, MgSO_4_•7H_2_O 1.17, NaH_2_PO_4_•2H_2_O 1.2, pyruvate 2.0, EDTA 2.00, MOPS 3.0, and glucose 5.0). Under Ca^2+^-free conditions, vessels underwent the same step-wise increases in transmural pressure (20 to 100 mmHg) and passive diameter (dia_max_) was recorded at each pressure. Myogenic tone was calculated as: myogenic tone (%)  =  (dia_max_ – dia_response_)/dia_max_ x 100, where dia_response_ is the diameter at a given pressure. For agonist-induced vasoconstriction, responses were compared to passive diameter under Ca^2+^-free conditions at dia_45mmHg_. Agonist-induced responses were calculated as: tone (%)  =  dia_45mmHg_ –dia_response_)/dia_45mmHg_ x 100, where dia_response_ is the diameter at a given concentration of the drug.

### Inflammatory responses

Mice were sacrificed at 64 hours post-MI (or sham controls) during their wake phase (ZT19), by isoflurane (4%) and cervical dislocation. Blood was collected for cytokine measurements, as previously described^23^. Briefly, plasma cytokines were quantified using the mouse cytometric bead array flex set (BD Biosciences) on an Accuri C6 flow cytometer (BD Biosciences), in accordance with the manufacturers instructions. Hearts were also collected from these mice and used for PCR of inflammasome genes, as described below.

### Heart mRNA and quantitative real-time polymerase chain reaction (RT-PCR)

Cardiac mRNA was purified using TRIzol (Invitrogen) and amplified by PCR as previously described^12,21,23,24^. Briefly, RT-PCR was performed using the Power SYBR Green RNA-to-CT 1-step kit (Thermo Fisher Scientific) on a ViiA7 real-time PCR system (Life Technologies) under the following protocol: reverse transcription, 48 °C for 30 min, 95 °C for 10 min for 1 cycle, amplification at 95 °C for 15 sec, 60 °C for 1 min for 40 cycles, followed by hold at room temperature. Primers: *NLR family pyrin domain containing 3* (*Nlrp3*) fwd (5′-CATGTTGCCTGTTCTTCCAGAC-3′), rev (5′-CGGTTGGTGCTTAGACTTGAGA-3′); *interleukin 1β* (*Il-1β)* fwd (5′-TGGGCCTCAAAGGAAAGAAT-3′), rev (5′-TGGGTATTGCTTGGGATCCA-3′); *histone* fwd (5′-GCAAGAGTGCGCCCTCTACTG-3′), rev (5′-GGCCTCACTTGCCTCCTGCAA-3′). RT-PCR was normalized to *histone* using the ΔΔCT method.

### mPFC and Hippocampus RNA isolation

*Clock*^*∆19/∆19*^ or WT mice were sacrificed at time 0 (baseline, BL), 64 hours post-MI (MI), or 8 weeks post-MI (HF) by isoflurane overdose and decapitation. All mice were sacrificed at ZT19 (wake time) to avoid confounding circadian effects in gene expression. Brains were quickly removed and mPFC and hippocampus tissue were dissected from acute brain slices prepared as previously described^71,72^. Tissues were snap frozen in liquid nitrogen and stored at −80 °C until use. Total RNA was isolated using the RNeasy Plus Mini Kit (Qiagen), according to the manufacturer’s instructions. RNA quality and quantity were assessed by NanoDrop ND-1000 (260/280 ≥ 2; Thermo Scientific). A minimum of 4 samples per tissue per condition were collected, resulting in a total of 48 samples processed on Affymetrix microarrays, which were then analyzed as described below.

### Microarrays and Bioinformatics

Whole genome microarray analyses were performed on mPFC or hippocampal total RNA using the Affymetrix GeneChip Mouse Gene 2.0 ST array, which interrogates>30,000 coding and non-coding transcripts and>2,000 long intergenic non-coding transcripts. To perform bioinformatics analyses, we used GeneSpring GX v14.9 software (Agilent Technologies Inc.). To do this, raw.cel files were loaded into a project file under exon analysis and Affymetrix exon expression experiment type settings with a biological significance workflow analysis. Analyses were performed using MoGene-2_0-st_na36_mm10_2016-07-06 annotation technology. The intensity across all chips was normalized using the exon robust multiarray summarization algorithm on all probesets. Experiment parameters were defined for the sample groups and replicate structure under a group-level interpretation. Principal components analysis (PCA) clustering and log2 (normalized signal values) of hybridization controls were used to assess sample quality and group clustering. First, all entities were selected in the probeset filter parameter, and then all probesets were filtered by expression with a minimum cut-off of ≥60 raw fluorescence units. Using fold change analysis, a list of all genes with ≥1.3-fold change in expression in *Clock*^*Δ19/Δ19*^ versus WT sections were selected. Separate analyses were performed for BL, MI, or HF samples, and for samples taken from the mPFC or hippocampus. Differentially expressed genes at baseline were also interrogated for converging expression in HF. Lists generated were subjected to Gene Ontology (GO) pathway analysis, and we selected for genes that map to neuron pathways using the search terms neuron morphology, growth, signaling, cytoskeleton, calcium signaling, ion channels, or metabolism. Heat maps were generated to display differentially regulated gene cassettes of interest. Gene lists were also investigated using the Kyoto Encyclopedia of Genes and Genomes (KEGG) pathway analysis, and the Database for Annotation Visualization and Integrated Discovery (DAVID) Functional Annotation Tool (DAVID Bioinformatics 6.8, NIAID/NIH)^73^. All microarray data were deposited in the Gene Expression Omnibus database (GSE124670).

### Statistical analysis

All values are expressed as mean ± SEM. Echocardiography, hemodynamics and histology data were analyzed using two-way analysis of variance (ANOVA) followed by Tukey’s *post-hoc* analysis. Neuron morphology data were analyzed by two-way ANOVA followed by Bonferroni’s multiple comparison correction. Behavioural data were analyzed using a split-plot two-way ANOVA followed by Bonferroni’s multiple comparison correction. Myography data were analyzed using two-way ANOVA followed by Bonferroni’s multiple-comparison correction. Microarray gene expression data was analyzed by GeneSpring GX v14.9 software (Agilent Technologies Inc). *P*-values  < 0.05 were considered statistically significant. All values were analyzed in GraphPad Prism 6 statistical software and plotted in Prism 6 or Microsoft Excel.

## Supplementary information

Supplementary Figure S1.

Supplementary Dataset 1

## References

[1] Mozaffarian D (2015). Heart disease and stroke statistics-2015 update: a report from the American Heart Association. Circulation.

[2] Almeida OP (2012). Cognitive and brain changes associated with ischaemic heart disease and heart failure. Eur Heart J.

[3] Sauve MJ, Lewis WR, Blankenbiller M, Rickabaugh B, Pressler SJ (2009). Cognitive impairments in chronic heart failure: a case controlled study. J Card Fail.

[4] Koenig HG (2006). Depression outcome in inpatients with congestive heart failure. Arch Intern Med.

[5] Clarke JM, Hamer J, Shelton JR, Taylor S, Venning GR (1976). The rhythm of the normal human heart. Lancet.

[6] Millar-Craig MW, Bishop CN, Raftery EB (1978). Circadian variation of blood-pressure. Lancet.

[7] Martino T (2004). Day/night rhythms in gene expression of the normal murine heart. J Mol Med (Berl).

[8] Storch KF (2002). Extensive and divergent circadian gene expression in liver and heart. Nature.

[9] Martino TA (2007). Disturbed diurnal rhythm alters gene expression and exacerbates cardiovascular disease with rescue by resynchronization. Hypertension.

[10] Bray MS (2008). Disruption of the circadian clock within the cardiomyocyte influences myocardial contractile function, metabolism, and gene expression. Am J Physiol-Heart C.

[11] Young ME (2014). Cardiomyocyte-specific BMAL1 plays critical roles in metabolism, signaling, and maintenance of contractile function of the heart. J Biol Rhythms.

[12] Tsimakouridze EV (2012). Chronomics of pressure overload-induced cardiac hypertrophy in mice reveals altered day/night gene expression and biomarkers of heart disease. Chronobiol Int.

[13] Chalmers JA (2008). Diurnal profiling of neuroendocrine genes in murine heart, and shift in proopiomelanocortin gene expression with pressure-overload cardiac hypertrophy. J Mol Endocrinol.

[14] Chalmers JA (2008). Vascular circadian rhythms in a mouse vascular smooth muscle cell line (Movas-1). Am J Physiol Regul Integr Comp Physiol.

[15] Podobed P (2014). The day/night proteome in the murine heart. Am J Physiol Regul Integr Comp Physiol.

[16] Podobed PS, Alibhai FJ, Chow CW, Martino TA (2014). Circadian regulation of myocardial sarcomeric Titin-cap (Tcap, telethonin): identification of cardiac clock-controlled genes using open access bioinformatics data. PLoS One.

[17] Martino TA, Tata N, Bjarnason GA, Straume M, Sole MJ (2007). Diurnal protein expression in blood revealed by high throughput mass spectrometry proteomics and implications for translational medicine and body time of day. Am J Physiol Regul Integr Comp Physiol.

[18] Alibhai FJ, Tsimakouridze EV, Reitz CJ, Pyle WG, Martino TA (2015). Consequences of Circadian and Sleep Disturbances for the Cardiovascular System. Can J Cardiol.

[19] Martino TA, Sole MJ (2009). Molecular time: an often overlooked dimension to cardiovascular disease. Circ Res.

[20] Martino TA, Young ME (2015). Influence of the cardiomyocyte circadian clock on cardiac physiology and pathophysiology. J Biol Rhythms.

[21] Alibhai FJ (2017). Disrupting the key circadian regulator CLOCK leads to age-dependent cardiovascular disease. J Mol Cell Cardiol.

[22] Alibhai, F. J. *et al*. Female ClockDelta19/Delta19 Mice are Protected from the Development of Age-Dependent Cardiomyopathy. *Cardiovasc Res*, 10.1093/cvr/cvx185 (2017). 10.1093/cvr/cvx185 28927226

[23] Alibhai FJ (2014). Short-term disruption of diurnal rhythms after murine myocardial infarction adversely affects long-term myocardial structure and function. Circ Res.

[24] Bennardo M (2016). Day-night dependence of gene expression and inflammatory responses in the remodeling murine heart post-myocardial infarction. Am J Physiol Regul Integr Comp Physiol.

[25] Martino TA (2008). Circadian rhythm disorganization produces profound cardiovascular and renal disease in hamsters. Am J Physiol Regul Integr Comp Physiol.

[26] Martino TA (2011). The primary benefits of angiotensin-converting enzyme inhibition on cardiac remodeling occur during sleep time in murine pressure overload hypertrophy. J Am Coll Cardiol.

[27] Martino, T. A. & Young, M. E. *Circadian Medicine*. *Science*. http://science.sciencemag.org/content/354/6315/986/tab-e-letters, 2017).

[28] Mistry P, D. A., Kirshenbaum, L, Martino, TA. Cardiac Clocks and Preclinical Translation. *Heart Failure Clinics* In press, 10.1016/j.hfc.2017.05.002 (2017). 10.1016/j.hfc.2017.05.002 28865775

[29] Reitz CJ, Martino TA (2015). Disruption of Circadian Rhythms and Sleep on Critical Illness and the Impact on Cardiovascular Events. Curr Pharm Des.

[30] Sole MJ, Martino TA (2009). Diurnal physiology: core principles with application to the pathogenesis, diagnosis, prevention, and treatment of myocardial hypertrophy and failure. J Appl Physiol (1985).

[31] Tsimakouridze EV, Alibhai FJ, Martino TA (2015). Therapeutic applications of circadian rhythms for the cardiovascular system. Front Pharmacol.

[32] Durgan DJ, Young ME (2010). The cardiomyocyte circadian clock: emerging roles in health and disease. Circ Res.

[33] Roenneberg T, Merrow M (2005). Circadian clocks - the fall and rise of physiology. Nat Rev Mol Cell Biol.

[34] Reppert SM, Weaver DR (2002). Coordination of circadian timing in mammals. Nature.

[35] Vitaterna MH (1994). Mutagenesis and mapping of a mouse gene, Clock, essential for circadian behavior. Science.

[36] Gekakis N (1998). Role of the CLOCK protein in the mammalian circadian mechanism. Science.

[37] Durgan DJ (2011). Evidence suggesting that the cardiomyocyte circadian clock modulates responsiveness of the heart to hypertrophic stimuli in mice. Chronobiol Int.

[38] Musiek ES (2013). Circadian clock proteins regulate neuronal redox homeostasis and neurodegeneration. J Clin Invest.

[39] Kondratova AA, Dubrovsky YV, Antoch MP, Kondratov RV (2010). Circadian clock proteins control adaptation to novel environment and memory formation. Aging (Albany NY).

[40] Gibson EM, Wang C, Tjho S, Khattar N, Kriegsfeld LJ (2010). Experimental 'jet lag' inhibits adult neurogenesis and produces long-term cognitive deficits in female hamsters. PLoS One.

[41] Karatsoreos IN, Bhagat S, Bloss EB, Morrison JH, McEwen BS (2011). Disruption of circadian clocks has ramifications for metabolism, brain, and behavior. Proc Natl Acad Sci USA.

[42] Woo MA, Kumar R, Macey PM, Fonarow GC, Harper RM (2009). Brain injury in autonomic, emotional, and cognitive regulatory areas in patients with heart failure. J Card Fail.

[43] Schleifer SJ (1989). The nature and course of depression following myocardial infarction. Arch Intern Med.

[44] Hance M, Carney RM, Freedland KE, Skala J (1996). Depression in patients with coronary heart disease. A 12-month follow-up. Gen Hosp Psychiatry.

[45] Shapiro PA (2015). Management of depression after myocardial infarction. Curr Cardiol Rep.

[46] Joynt KE, Whellan DJ, O'Connor CM (2003). Depression and cardiovascular disease: mechanisms of interaction. Biol Psychiatry.

[47] Evonuk KS, Prabhu SD, Young ME, DeSilva TM (2017). Myocardial ischemia/reperfusion impairs neurogenesis and hippocampal-dependent learning and memory. Brain Behav Immun.

[48] Xie Z (2015). Smooth-muscle BMAL1 participates in blood pressure circadian rhythm regulation. J Clin Invest.

[49] Su W (2012). Altered clock gene expression and vascular smooth muscle diurnal contractile variations in type 2 diabetic db/db mice. Am J Physiol Heart Circ Physiol.

[50] Anea CB (2009). Vascular disease in mice with a dysfunctional circadian clock. Circulation.

[51] Douma, L. G. & Gumz, M. L. Circadian clock-mediated regulation of blood pressure. *Free Radic Biol Med*, 10.1016/j.freeradbiomed.2017.11.024 (2017). 10.1016/j.freeradbiomed.2017.11.024 PMC5910276 29198725

[52] Kroetsch JT, Bolz SS (2013). The TNF-alpha/sphingosine-1-phosphate signaling axis drives myogenic responsiveness in heart failure. J Vasc Res.

[53] Edenberg HJ (2004). Variations in GABRA2, encoding the alpha 2 subunit of the GABA(A) receptor, are associated with alcohol dependence and with brain oscillations. Am J Hum Genet.

[54] Arai Y (2005). Role of Fabp7, a downstream gene of Pax6, in the maintenance of neuroepithelial cells during early embryonic development of the rat cortex. J Neurosci.

[55] McKerracher L (1994). Identification of myelin-associated glycoprotein as a major myelin-derived inhibitor of neurite growth. Neuron.

[56] Philibert RA, Winfield SL, Sandhu HK, Martin BM, Ginns EI (2000). The structure and expression of the human neuroligin-3 gene. Gene.

[57] Chen H (2003). Mitofusins Mfn1 and Mfn2 coordinately regulate mitochondrial fusion and are essential for embryonic development. J Cell Biol.

[58] Khaper N (2018). Implications of disturbances in circadian rhythms for cardiovascular health: A new frontier in free radical biology. Free Radic Biol Med.

[59] Alibhai FJ (2018). Female ClockDelta19/Delta19 mice are protected from the development of age-dependent cardiomyopathy. Cardiovasc Res.

[60] Basu P (2015). Male-Specific Cardiac Dysfunction in CTP:Phosphoethanolamine Cytidylyltransferase (Pcyt2)-Deficient Mice. Mol Cell Biol.

[61] Pyle WG, Martino TA (2018). Circadian rhythms influence cardiovascular disease differently in males and females: role of sex and gender. Current Opinion in Physiology.

[62] Buxton OM (2012). Sleep disruption due to hospital noises: a prospective evaluation. Ann Intern Med.

[63] Drouot X, Cabello B, d'Ortho MP, Brochard L (2008). Sleep in the intensive care unit. Sleep Med Rev.

[64] Louth EL, Luctkar HD, Heney KA, Bailey CDC (2018). Developmental ethanol exposure alters the morphology of mouse prefrontal neurons in a layer-specific manner. Brain Res.

[65] Louth EL, Sutton CD, Mendell AL, MacLusky NJ, Bailey CDC (2017). Imaging Neurons within Thick Brain Sections Using the Golgi-Cox Method. J Vis Exp.

[66] Mitchnick KA, Creighton S, O'Hara M, Kalisch BE, Winters BD (2015). Differential contributions of de novo and maintenance DNA methyltransferases to object memory processing in the rat hippocampus and perirhinal cortex-a double dissociation. Eur J Neurosci.

[67] Bartko SJ, Winters BD, Cowell RA, Saksida LM, Bussey TJ (2007). Perceptual functions of perirhinal cortex in rats: zero-delay object recognition and simultaneous oddity discriminations. J Neurosci.

[68] Cloke JM (2016). A Novel Multisensory Integration Task Reveals Robust Deficits in Rodent Models of Schizophrenia: Converging Evidence for Remediation via Nicotinic Receptor Stimulation of Inhibitory Transmission in the Prefrontal Cortex. J Neurosci.

[69] Meissner, A. *et al*. Tumor Necrosis Factor-alpha Underlies Loss of Cortical Dendritic Spine Density in a Mouse Model of Congestive Heart Failure. *J Am Heart Assoc* **4**, 10.1161/JAHA.115.001920 (2015). 10.1161/JAHA.115.001920 PMC4599420 25948533

[70] Yang J (2012). Proximal cerebral arteries develop myogenic responsiveness in heart failure via tumor necrosis factor-alpha-dependent activation of sphingosine-1-phosphate signaling. Circulation.

[71] Chung BY, Bignell W, Jacklin DL, Winters BD, Bailey CD (2016). Postsynaptic nicotinic acetylcholine receptors facilitate excitation of developing CA1 pyramidal neurons. J Neurophysiol.

[72] Louth, E. L., Bignell, W., Taylor, C. L. & Bailey, C. D. Developmental Ethanol Exposure Leads to Long-Term Deficits in Attention and Its Underlying Prefrontal Circuitry. *eNeuro* **3**, 10.1523/ENEURO.0267-16.2016 (2016). 10.1523/ENEURO.0267-16.2016 PMC5099605 27844059

[73] Huang da W, Sherman BT, Lempicki RA (2009). Systematic and integrative analysis of large gene lists using DAVID bioinformatics resources. Nat Protoc.

