# Supplementary material for: The Clock Mechanism Influences Neurobiology and Adaptations to Heart Failure in Clock∆19/∆19 Mice With Implications for Circadian Medicine
Source: Sci Rep. 2019 Mar 21;9:4994. doi: 10.1038/s41598-019-41469-7 (PMC6428811; doi:10.1038/s41598-019-41469-7)
Supplement: Supplementary file 1 — Supplementary Figure S1. [file 41598_2019_41469_MOESM1_ESM.docx]

**The Clock Mechanism Influences Neurobiology and Adaptations to Heart Failure in *Clock^∆19/∆19^* Mice With Implications for Circadian Medicine**

Austin T.H. Duong^1+^, Cristine J. Reitz^1+^, Emma L. Louth^1^, Samantha D. Creighton^2^, Mina Rasouli^1^, Ashley Zwaiman^1^, Jeffrey T. Kroetsch^3^, Steffen-Sebastian Bolz^3^, Boyer D. Winters^2^, Craig D.C. Bailey^1^*, Tami A. Martino^1^*

^1^Centre for Cardiovascular Investigations, Biomedical Sciences, University of Guelph, Guelph, Ontario, Canada, ^2^Department of Psychology, University of Guelph, Guelph, Ontario, Canada, ^3^Department of Physiology, University of Toronto, Toronto, Ontario, Canada. ^+^Authors contributed equally.

*Correspondence to:

Dr. Tami A. Martino, PhD, Centre for Cardiovascular Investigations, Biomedical Sciences / OVC Room 1646B, University of Guelph, Guelph, Ontario, Canada, N1G 2W1 E-mail: [tmartino@uoguelph.ca](mailto:tmartino@uoguelph.ca)

Dr. Craig D.C. Bailey, PhD, Biomedical Sciences / OVC, University of Guelph, Guelph, Ontario, Canada, N1G 2W1 E-mail: [baileyc@uoguelph.ca](mailto:baileyc@uoguelph.ca)

**Supplementary Figure S1**

**a**

**b**

**Supplementary Figure S1.** Early systemic responses are similar in the hearts of *Clock^Δ19/Δ19^* and WT mice post-MI. **(a)** Cardiac mRNA levels of the *Nlrp3* inflammasome and associated cytokines *Il-1β* and *Il-18*, and **(b)** plasma cytokine levels. 64h post-MI, n>4, values are mean ± SEM.
